# Phylogenomic analysis of the family *Peptostreptococcaceae* (*Clostridium* cluster XI) and proposal for reclassification of *Clostridium litorale* (Fendrich *et al.* 1991) and *Eubacterium acidaminophilum* (Zindel *et al.* 1989) as *Peptoclostridium litorale* gen. nov. comb. nov. and *Peptoclostridium acidaminophilum* comb. nov.

**DOI:** 10.1099/ijsem.0.001548

**Published:** 2016-12

**Authors:** Michael Y. Galperin, Vyacheslav Brover, Igor Tolstoy, Natalya Yutin

**Affiliations:** National Center for Biotechnology Information, National Library of Medicine, National Institutes of Health, Bethesda, Maryland 20894, USA

**Keywords:** Taxonomy, Gram-positive bacteria, anaerobes, genome analysis, 16S rRNA

## Abstract

In 1994, analyses of clostridial 16S rRNA gene sequences led to the assignment of 18 species to *Clostridium* cluster XI, separating them from *Clostridium sensu stricto* (*Clostridium* cluster I). Subsequently, most cluster XI species have been assigned to the family *Peptostreptococcaceae* with some species being reassigned to new genera. However, several misclassified *Clostridium* species remained, creating a taxonomic conundrum and confusion regarding their status. Here, we have re-examined the phylogeny of cluster XI species by comparing the 16S rRNA gene-based trees with protein- and genome-based trees, where available. The resulting phylogeny of the *Peptostreptococcaceae* was consistent with the recent proposals on creating seven new genera within this family. This analysis also revealed a tight clustering of *Clostridium litorale* and *Eubacterium acidaminophilum*. Based on these data, we propose reassigning these two organisms to the new genus *Peptoclostridium* as *Peptoclostridium litorale* gen. nov. comb. nov. (the type species of the genus) and *Peptoclostridium acidaminophilum* comb. nov., respectively. As correctly noted in the original publications, the genera *Acetoanaerobium* and *Proteocatella* also fall within cluster XI, and can be assigned to the *Peptostreptococcaceae. Clostridium sticklandii*, which falls within radiation of genus *Acetoanaerobium*, is proposed to be reclassified as *Acetoanaerobium sticklandii* comb. nov. The remaining misnamed members of the *Peptostreptococcaceae*, [*Clostridium*] *hiranonis*, [*Clostridium*] *paradoxum* and [*Clostridium*] *thermoalcaliphilum*, still remain to be properly classified.

In the past, obligately anaerobic spore-forming bacteria that stained Gram-positive were often assigned to the genus *Clostridium*, which resulted in a single genus with more than 200 validly named species with vastly different properties, including proteolytic and cellulosolytic bacteria, some pathogenic and some benign (Parte, 2014). In 1994, Collins and colleagues used 16S rRNA gene sequences to divide clostridial species into 19 clusters; each cluster included several proposed genera and corresponded to a family-level taxon (Collins *et al.*, 1994). In subsequent studies, most of those groupings have been confirmed and the taxonomy of many former *Clostridium* species has been streamlined by reassigning them to new genera. It has become clear that only *Clostridium* cluster I species (*Clostridium sensu stricto*) are sufficiently close to the type species *Clostridium butyricum* to qualify as members of the same genus. This view has been reinforced by the recent work by Lawson & Rainey (2016), who removed from the genus *Clostridium* even cluster II species, *Clostridium histolyticum*, *Clostridium limosum* and *Clostridium proteolyticum*, which are the closest relatives of cluster I, and reassigned them to the new genus *Hathewaya*. In the clostridial classification update in the latest edition of Bergey’s, many former *Clostridium* species have been moved to families *Lachnospiraceae*, *Peptostreptococcaceae* and *Ruminococcaceae* in the order *Clostridiales* and to the family *Erysipelotrichaceae* in the class *Erysipelotrichia* (Ludwig *et al.*, 2009a). However, most of these organisms still retained the ‘*Clostridium*’ name (Rainey *et al.*, 2009; Parte, 2014), resulting in a taxonomic and nomenclature conundrum, where the *Clostridium* species designation did not necessarily indicate a close relationship to the type species *Clostridium butyricum* and such *Clostridium* *sensu stricto* organisms as *Clostridium botulinum* and *Clostridium tetani*. To deal with this conundrum, the NCBI Taxonomy Database and SILVA database display such misnamed organisms as [*Clostridium*] species (Federhen, 2012, 2015; Yilmaz *et al.*, 2014); for clarity, this approach is also used in this work. This designation helps in highlighting the problem but obviously does not resolve it.

In 2013, Yutin and Galperin proposed to resolve the inconsistency of having 15 validly described *Clostridium* species in the family *Peptostreptococcaceae* by tentatively assigning them, along with *Eubacterium tenue* and *Eubacterium yurii*, to the new genus *Peptoclostridium* (Yutin & Galperin, 2013). They have argued that, although imperfect and tentative, such a solution was still better than either listing these organisms as [*Clostridium*] species or preserving the *Clostridium* species name for the bacteria that clearly do not belong to the genus *Clostridium* or even the family *Clostridiaceae*. Unfortunately, the proposal to include all diverse species into a single genus proved to be unsatisfactory. In addition, this proposal did not comply with the Bacteriological Code (Parker *et al.*, 2015), and its partial adoption by some databases only increased the confusion in clostridial nomenclature.

Recently, this nomenclature problem was partly resolved by three papers that reassigned 10 of those 15 [*Clostridium*] species to the new genera. First, Gerritsen and colleagues proposed creating four new genera *Romboutsia*, *Intestinibacter*, *Terrisporobacter* and *Asaccharospora*, which accommodated five former [*Clostridium*] species: *Clostridium bartlettii*, *Clostridium glycolicum*, *Clostridium irregulare*, *Clostridium lituseburense* and *Clostridium mayombei* (Gerritsen *et al.*, 2014). Earlier this year, Sasi Jyothsna and colleagues proposed creation of two more genera, *Paraclostridium* and *Paeniclostridium*, to accommodate three more such species: *Clostridium bifermentans*, *Clostridium ghonii* and *Clostridium sordellii* (Sasi Jyothsna *et al.*, 2016). Finally, Lawson and colleagues proposed reassigning [*Clostridium*] *difficile* and *Clostridium mangenotii* to the new genus *Clostridioides* (Lawson *et al.*, 2016). Although these studies substantially streamlined the nomenclature of the family *Peptostreptococcaceae*, several members of this family remain listed as [*Clostridium*] or [*Eubacterium*] species. Here, we report a re-analysis of the available sequence data for the members of the family *Peptostreptococcaceae*, confirm the groupings proposed by Gerritsen *et al.* (2014), Sasi Jyothsna *et al.* (2016) and Lawson *et al.* (2016) and propose re-assigning [*Clostridium*] *litorale* and [*Eubacterium*] *acidaminophilum* to the new genus *Peptoclostridium*. We additionally propose transfer of the genera *Acetoanaerobium* and *Proteocatella* to the *Peptostreptococcaceae* and reclassification of [*Clostridium*] *sticklandii* as *Acetoanaerobium sticklandii*.

The original description of the clostridial cluster XI (Collins *et al.*, 1994) included 19 validly described species assigned to eight proposed genera with an additional genus reserved for ‘*Clostridium aminobutyricum*’ (not validly described, currently listed as *Clostridium* sp. DSM 2634=ATCC 13726). Subsequent analyses of 16S rRNA gene sequences showed that members of three proposed genera (listed as genera 6, 7 and 8 in Collins *et al.*, 1994), namely ‘*C. aminobutyricum’*, *Clostridium felsineum*, *Clostridium formicaceticum*, and *Clostridium halophilum*, along with the more recently described *Clostridium caminithermale*, form a relatively compact separate group that also includes *Clostridium aceticum* and *Anaerovirgula multivorans* (Brisbarre *et al.*, 2003; Chen *et al.*, 2006; Pikuta *et al.*, 2006; Gerritsen *et al.*, 2014; Poehlein *et al.*, 2015; Lawson *et al.*, 2016). In the 2009 edition of Bergey’s, these species were assigned to *Clostridiaceae* 2, a sister group to *Peptostreptococcaceae* (Ludwig *et al.*, 2009b), and are listed the same way in the SILVA database (Yilmaz *et al.*, 2014). In our hands, 16S rRNA gene sequences from these organisms also formed a separate group. Also, aside from the genome of *C. aceticum* (Poehlein *et al.*, 2015), there was almost no sequence data to verify the 16S rRNA gene-based tree. Therefore, these organisms were excluded from further analysis. The phylogeny of the remaining members of cluster XI and the *Peptostreptococcaceae* was analysed using 16S rRNA gene-based, protein-based and whole genome-based phylogenetic trees.

The 16S rRNA gene trees reconstructed using neighbour-joining and maximum likelihood methods (Figs 1 and S1, available in the online Supplementary Material) were similar to those reported previously (Collins *et al.*, 1994; Chen, 2006; Gerritsen *et al.*, 2014; Lawson *et al.*, 2016; Sasi Jyothsna *et al.*, 2016). They showed clear groupings of the species within the genera *Filifactor*, *Peptostreptococcus* and *Tepidibacter*. These trees also confirmed grouping of the former [*Clostridium*] *glycolicus* and *Clostridium mayombei*, which had been reassigned to the genus *Terrisporobacter* (Gerritsen *et al.*, 2014), of the former *C*.*ghonii* with *C. sordellii*, reassigned to the genus *Paeniclostridium* (Sasi Jyothsna *et al.*, 2016), and of *C*.*difficile* and *C. mangenotii*, recently reassigned to the genus *Clostridioides* (Lawson *et al.*, 2016). Given the reclassification of the former *C. bartlettii* as *Intestinibacter bartlettii*; *C. irregulare* as *Asaccharospora irregularis*; *C. lituseburense* as *Romboutsia lituseburensis* (Gerritsen *et al.*, 2014); and of *C. bifermentans* as *Paraclostridium bifermentans* (Sasi Jyothsna *et al.*, 2016), only five validly described members of the family *Peptostreptococcaceae* (four from the original cluster XI and *Clostridium hiranonis*) remained listed as [*Clostridium*] species. Finally, in accordance with the original publications (Pikuta *et al.*, 2009; Bes *et al.*, 2015), the 16S rRNA gene tree showed that members of the genera *Acetoanaerobium* (*Acetoanaerobium noterae* and *Acetoanaerobium pronyense*) and *Proteocatella* (*Proteocatella sphenisci*) also fall within cluster XI and family *Peptostreptococcaceae*. We therefore formally propose reassigning genera *Acetoanaerobium* and *Proteocatella* to the family *Peptostreptococcaceae*.

**Fig. 1.**
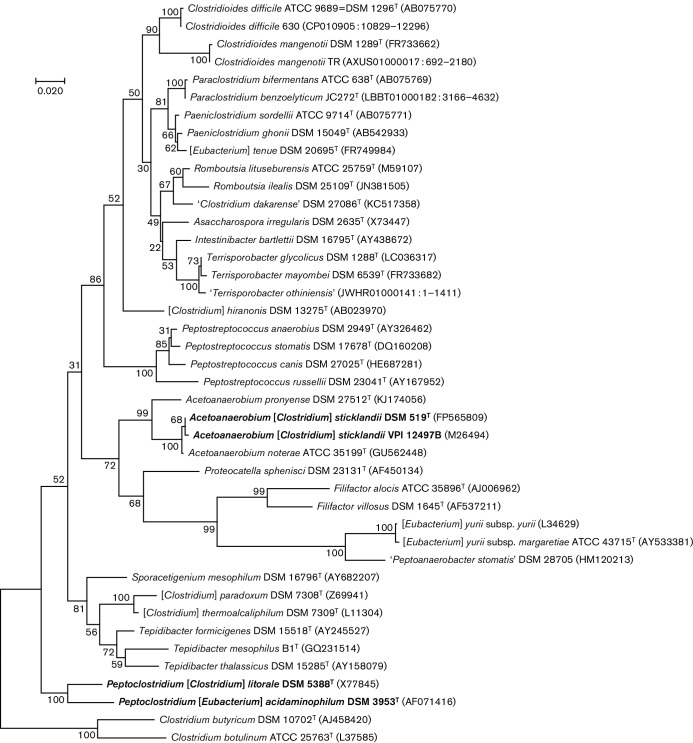
16S rRNA gene-based phylogenetic tree of the family *Peptostreptococcaceae.* The sequences from type strains (indicated with superscript T) were used and listed under their DSM accession numbers, where available. GenBank accession numbers are listed in parentheses. For *Clostridioides difficile* 630, *Clostridioides mangenotii* TR, *Paraclostridium benzoelyticum* JC272^T^, and ‘*Terrisporobacter othiniensis*’, 16S rRNA gene sequences were taken from the respective genomic entries. Quotation marks indicate the organisms whose names have not yet been validly published. The organisms that this work proposes to be renamed are indicated in boldtype. The sequences were aligned using muscle (Edgar, 2004) and the tree was inferred using the maximum-likelihood method based on the Tamura–Nei model (Tamura & Nei, 1993) as implemented in mega6 (Tamura *et al.*, 2013); for the initial neighbour-joining tree, see Fig. S1. The tree was rooted using sequences from *C. butyricum* and *C. botulinum*, members of *Clostridium sensu stricto*.

The phylogenetic trees generated using 16S rRNA gene sequences also showed that *C. litorale* and *E. acidaminophilum* formed a well-supported separate branch (Fig. 1), which has also been seen in previous reports (Baena *et al.*, 1999; Pikuta *et al.*, 2009; Gerritsen *et al.*, 2014; Rainey *et al.*, 2015; Lawson *et al.*, 2016), see e.g. Fig. 143 in Wade (2009). This association suggested that these two species might qualify for inclusion into the same genus. Indeed, 16S rRNA genes of *C. litorale* and *E. acidaminophilum* share 94 % sequence identity over 1500 bases, which is close to the threshold for a single genus suggested by Yarza *et al.* (2008, 2014) and supported by Tindall *et al.* (2010). Previously, Collins *et al.* (1994) assigned *C. litorale* to a separate genus in cluster XI. Later, Baena *et al.* (1999) determined the 16S rRNA gene sequence of *E. acidaminophilum*, showed its tight clustering with *C. litorale*, and recommended creating a new genus to accommodate these two species. We wanted an independent means of verifying the close relation between C.*litorale* and *E. acidaminophilum* and checking the consistency of other groupings seen on the 16S rRNA gene tree. To this end, we reconstructed a concatenated alignment of 50 widespread ribosomal proteins and also alignments for DNA-directed RNA polymerase beta subunit (RpoB) and DNA gyrase subunit B (GyrB) from various members of *Peptostreptococcaceae*. These alignments were sufficiently long to allow fine mapping of phylogenetic relations and analyse deep phylogenetic lineages (Yutin *et al.*, 2012; Yutin & Galperin, 2013). However, the use of such trees in clostridial phylogeny is limited by the fact that protein sequences are currently available only for a limited number of strains (Table S1). Fortunately, genome sequences of *C. litorale* and *E. acidaminophilum* have already been made available by Poehlein *et al.* (2014a, b).

The ribosomal proteins-based tree (Fig. 2) confirmed the close relationship between *C. litorale* and *E. acidaminophilum*, as well as the groupings proposed previously by Gerritsen *et al.* (2014), Sasi Jyothsna *et al.* (2016) and Lawson *et al.* (2016). The RpoB and GyrB trees (Fig. S2) had similar topologies and also showed *C. litorale* and *E. acidaminophilum* branching together. In addition, grouping of *C. litorale* and *E. acidaminophilum* could be seen on the tree built from whole-genome alignments of members of *Peptostreptococcaceae* (Fig. S3a), using the approaches developed by Goris *et al.* (2007), Deloger *et al.* (2009) and Varghese *et al.* (2015). Visualization of this tree on a two-dimensional plot using principal component analysis (Fig. S3b) also showed *C. litorale* and *E. acidaminophilum* clustering separately from other species.

**Fig. 2.**
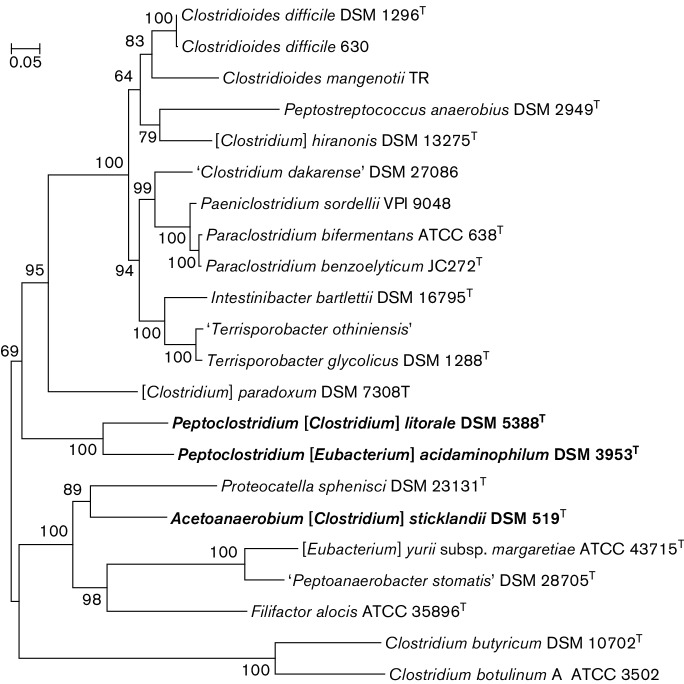
Ribosomal protein-based tree of the members of *Peptostreptococcaceae*. A maximum-likelihood tree was built using the PhyML program (Guindon *et al.*, 2010) from a concatenated alignment of 50 ribosomal proteins (L1–L7, L9–L11, L13–L24, L27–L29, L31–L36 and S2–S20), with a total of 6269 aligned positions, as described previously (Yutin *et al.*, 2012; Yutin & Galperin, 2013). The organisms proposed for renaming are indicated in boldtype. The names of the organisms that have not been validly published are in quotation marks. The tree was rooted using sequences from *C. butyricum* and *C. botulinum*.

In addition to sequence information, *C. litorale* and *E. acidaminophilum* share several important physiological properties, including the ability to grow on betaine and such amino acids as glycine and serine, metabolizing them via Stickland reaction (Dietrichs *et al.*, 1991), and the lack of utilization of carbohydrates. These traits are usually not seen in other representatives of *Peptostreptococcaceae* (Table S2). The most conspicuous difference between the two species is that *C. litorale* is a spore former, whereas *E. acidaminophilum* is not (Zindel *et al.*, 1988; Fendrich *et al.*, 1990). However, *E. acidaminophilum* encodes almost as many proteins as *C. litorale* (Table S1), including some very similar core sporulation proteins (Galperin *et al.*, 2012), see Table S3. Therefore, the lack of sporulation in *E. acidaminophilum* is likely due to a relatively recent loss of certain sporulation genes in that particular lineage. Based on these data, we propose reclassification of *C. litorale* and *E. acidaminophilum* into the new genus *Peptoclostridium* as *Peptoclostridium litorale* gen. nov. comb. nov. and *Peptoclostridium acidaminophilum* comb. nov., respectively.

It must be noted that the current proposal of genus *Peptoclostridium* is substantially different from the one put forward previously by Yutin & Galperin (2013), which included all 15 validly described [*Clostridium*] members of the *Peptostreptococcaceae*. As noted above, 10 out of those 15 [*Clostridium*] species have already been re-assigned to new genera within *Peptostreptococcaceae* (Gerritsen *et al.*, 2014; Lawson *et al.*, 2016; Sasi Jyothsna *et al.*, 2016). The proposed renaming of [*Clostridium*] *sticklandii* is discussed below. Two other species, *Clostridium paradoxum* and *Clostridium thermoalcaliphilum*, consistently cluster together and are weakly linked to *Tepidibacter* species (see Figs 1 and 2 and Gerritsen *et al.* (2014); Sasi Jyothsna *et al.*, 2016); they might have to be reassigned to a separate genus. *C. hiranonis* does not fit into any of the current genera and will probably have to be elevated to the genus level as well. Of the other [*Eubacterium*] members of the *Peptostreptococcaceae*, *E. tenue* is closely related to *Paeniclostridium ghonii* and *Paeniclostridium sordellii* (Wade, 2009; Sasi Jyothsna *et al.*, 2016) and clearly falls within the genus *Paeniclostridium*. The misnamed *E. yurii* fits into the recently proposed genus *Peptoanaerobacter* (Sizova *et al.*, 2015). ‘*Clostridium dakarense*’ (Lo *et al.*, 2013) is related to the members of genus *Romboutsia* and could be assigned to that genus (Fig. 1).

Among the [*Clostridium*] species that have not been validly described or deposited in microbial culture databases, ‘*Clostridium venationis*’ (16S rRNA gene GenBank accession number EU089966, 99 % identical to that of the type strain of *C. mangenotii*) is a candidate for inclusion into the genus *Clostridioides* (Fig. 1). According to the GenBank entry, this organism was a psychrotolerant, spore-forming, proteolytic bacterium isolated from a meat processing facility. A similar organism has been reportedly isolated from an anaerobically enriched uranium-contaminated soil sediment (Yang *et al.*, 2012). These two isolates substantially expand the ecological range of *Clostridioides* species. Of other [*Clostridium*] species known only by their 16S rRNA gene entries, ‘*Clostridium maritimum*’ (EU089965) and ‘*Clostridium ruminantium*’ (EU089964 and KJ722512) fall within the genus *Romboutsia*, while ‘*Clostridium metallolevans*’ (DQ133569, Meyer *et al.*, 2007) belongs to *Terrisporobacter* (Fig. 1).

It has been previously noted that [*Clostridium*] *sticklandii* strain DSM 519^T^ is closely related to *C. difficile* (Stadtman & McClung, 1957; Fonknechten *et al.*, 2010). However, on the 16S rRNA gene-based tree, this strain falls within the radiation of the genus *Acetoanaerobium* and next to the *Proteocatella* and *Filifactor* branches (Fig. 1 and Bes *et al.*, 2015; Scaria *et al.*, 2015). Accordingly, on the ribosomal protein tree that did not have any representatives of *Acetoanaerobium*, it clustered with *Proteocatella sphenisci* (Fig. 2). Therefore, we formally propose reclassification of [*Clostridium*] *sticklandii* as *Acetoanaerobium sticklandii* comb. nov. It should be noted that the assignment of the genus *Acetoanaerobium* to family *Peptostreptococcaceae* eliminates the need for Family XIX *Incertae Sedis*, created in the second edition of Bergey’s (Ludwig *et al.*, 2009a). A summary of the proposed name changes and an updated nomenclature of the family *Peptostreptococcaceae* are provided in Tables S4 and S5.

## Description of *Peptoclostridium* gen. nov.

*Peptoclostridium* [Pep.to.clos.tri′di.um. Gr. v. *peptô* digest; N.L. neut. dim. n. *Clostridium* a bacterial genus name (from Gr. n. *klôstêr* a spindle); N.L. neut. dim. n. *Peptoclostridium* the digesting clostridium].

Obligately anaerobic, motile, straight or slightly curved rods, 0.5–1.5×2–8 µm, metabolizing amino acids and oligopeptides but not carbohydrates. Gram-staining variable, Gram-positive-type cell wall contains *meso*-diaminopimelate. Cells grow at 15–40 °C, no growth at 42 °C; pH range is from 6.5 to 8.4, optimum pH is 7.1–7.4, can grow in defined media containing biotin using glycine or serine as sole carbon and energy source. Betaine and sarcosine (*N*-methylglycine) can be used as carbon and energy sources in the presence of electron donors, such as H_2_ or amino acids alanine, leucine, isoleucine, valine or phenylalanine. Utilization of glycine is via Stickland reaction catalysed by the glycine reductase complex and requires selenium (Dietrichs *et al.*, 1991). Glycine is metabolized to acetate, CO_2_ and NH_3_; serine is metabolized to acetate, ethanol, CO_2_, H_2_ and NH_3_. In complex media, acetate and butyrate are the major fermentation products. Growth is stimulated by low amounts of NaCl but completely inhibited by 6 % NaCl. Oxidase and catalase negative. Sulfate, thiosulfate and nitrate are not reduced. Cultured representatives of *Peptoclostridium* have been isolated from anaerobic mud in wastewater and marine sediments. Metagenomics analyses detected 16S rRNA genes from potential members of this genus in microbial consortia performing anaerobic dechlorination of hexachlorobenzene and polychlorinated biphenyls and dibenzofurans (Yoshida *et al.*, 2005; Ho & Liu, 2011; Zhou *et al.*, 2015), an anaerobic cellulolytic microbial consortium from mangrove soil (Gao *et al.*, 2014), as well as in a methanogenic bioreactor degrading terephthalate (Nobu *et al.*, 2015). The G+C content of genomic DNA ranges from 41.3 to 44.0 mol%. The type species is *Peptoclostridium litorale* (basonym *Clostridium litorale* Fendrich *et al.* 1991).

## Description of *Peptoclostridium litorale* comb. nov.

*Peptoclostridium litorale* (li.to.ra′le. L. neut. adj. *litorale* coastal, referring to the source of the organism).

Basonym: *Clostridium litorale* Fendrich *et al.* 1991.

The description of *Peptoclostridium litorale* is identical to that provided for *Clostridium litorale* (Fendrich *et al.*, 1990; Rainey *et al.*, 2009). In addition to those described for the genus, has the following distinguishing properties. Cells stain Gram-negative. Form ovoid subterminal spores 1.5–2.0 µm in diameter. Colonies are brown, circular, with irregular margins.

The type strain W6^T^=ATCC 49638^T^=DSM 5388^T^ was isolated from anoxic marine sediment near the coast of the Jade Bay (Jadebusen) of the North Sea in Germany (Fendrich *et al.*, 1990). Sequence data from a whole-genome sequencing project (Poehlein *et al.*, 2014a) are available in GenBank accession no. JJMM01000000. The G+C content of the type strain chromosomal DNA was originally reported as 26.1 mol% (Fendrich *et al.*, 1990) but genome sequencing gave a much higher figure of 41.3 mol% (Poehlein *et al.*, 2014a).

## Description of *Peptoclostridium acidaminophilum* comb. nov.

*Peptoclostridium acidaminophilum* (a.cid.a.mi.no′phi.lum. N.L. neut. adj. *acidaminophilum* loving amino acids).

Basonym: *Eubacteriumacidaminophilum* Zindel *et al.* 1989.

The description of *Peptoclostridium acidaminophilum* is identical to that for *Eubacterium acidaminophilum* (Zindel *et al.*, 1988; Rainey *et al.*, 2009). In addition to those described for the genus, has the following distinguishing properties. Cells stain Gram-positive but behave Gram-negative in the KOH test. Spores are not observed. At least 1–2 mM NaCl are required for growth. Can utilize glycylglycine, glycylglycylglycine, glutathione and hydantoic acid.

The type strain al-2^T^=ATCC 49065^T^=DSM 3953^T^ was isolated from anaerobic black mud from a waste water ditch near Konstanz, Germany (Zindel *et al.*, 1988). The complete genomic sequence of the type strain has been determined (Poehlein *et al.*, 2014b) and is available in GenBank accession no. CP007452. The G+C content of the type strain chromosomal DNA is 44.0 mol%.

## Description of *Acetoanaerobium sticklandii* comb. nov.

*Acetoanaerobium sticklandii* (stick.lan′di.i N.L. gen. masc. n. *sticklandii* named after British biochemist Leonard Hubert Stickland, who described a key reaction in clostridial amino acid metabolism).

Basonym: *Clostridium sticklandii* Stadtman & McClung (1957) (Approved Lists 1980).

The description of *Acetoanaerobium sticklandii* is identical to that provided earlier for *Clostridiumsticklandii* (Stadtman & McClung, 1957; Rainey *et al.*, 2009). The type strain is ATCC 12662^T^=DSM 519^T^=JCM 1433^T^, isolated from black mud from the east shore of San Francisco Bay (Stadtman & Barker, 1951). A detailed comparison of this strain with *Acetoanaerobium noterae* NOT-3^T^ (=ATCC 35199^T^) and *Acetoanaerobium pronyense* ST07-YE^T^ (=DSM 27512^T^) has been published (Bes *et al.*, 2015). The complete genome sequence of the type strain is available (Fonknechten *et al.*, 2010); its G+C content is 33.3 mol%.

## Supplementary Data

842 Supplementary File 1
