# Supplementary material for: Phylogenomic analysis of the family Peptostreptococcaceae (Clostridium cluster XI) and proposal for reclassification of Clostridium litorale (Fendrich et al. 1991) and Eubacterium acidaminophilum (Zindel et al. 1989) as Peptoclostridium litorale gen. nov. comb. nov. and Peptoclostridium acidaminophilum comb. nov
Source: Int J Syst Evol Microbiol. 2016 Dec;66(12):5506–13. doi: 10.1099/ijsem.0.001548 (PMC5244501; doi:10.1099/ijsem.0.001548)
Supplement: Supplementary File 1 [file ijsem-66-1548-s001.pdf]

Supplementary Material

to "Phylogenomic analysis of the family *Peptostreptococcaceae* (Clostridium cluster XI)…"

by Michael Y. Galperin, Vyacheslav Brover, Igor Tolstoy, and Natalya Yutin.

Phylogenetic methods

1. 16S rRNA gene-based tree

The 16S rRNA gene sequences were obtained from GenBank (Benson *et al.*, 2015), based on the entries for type strains as listed in LPSN, StrainInfo, and SILVA databases (Parte, 2014; Verslyppe *et al.*, 2014; Yilmaz *et al.*, 2014) and referred to by the NCBI RefSeq Targeted Loci project (Federhen, 2015). The last database access date was July 1st, 2016. Sequences were aligned with MUSCLE (Edgar, 2004) as implemented in the MEGA6 software suite (Tamura *et al.*, 2013) and the phylogenetic tree on Figure 1 was constructed by applying the maximum likelihood method to the initial tree obtained using neighbor-joining algorithm (Figure S1).

2. Protein-based trees

Protein-based and whole-genome-based trees relied on the complete genomes and genomic DNA assemblies of various members of the *Peptostreptococcaceae* and protein sequences translated from them (Table S1). An alignment of 50 ribosomal proteins was constructed as described earlier (Yutin *et al.*, 2012; Yutin & Galperin, 2013), using concatenated sequences of L1-L7, L9-L11, L13-L24, L27-L29, L31-L36, and S2-S20 proteins from each organism (including new translations of the L36 proteins that had been missed in the genome annotations of [*Clostridium*] *hiranonis* DSM 13275^{T} and [*Clostridium*] *litorale* DSM 5388^{T}). Ribosomal protein sequences were aligned by MUSCLE (Edgar, 2004), the alignments were concatenated, and the resulting 6,269-position alignment was used for phylogenetic reconstruction. The phylogenetic tree was constructed using the PhyML program (Guindon *et al.*, 2010), the latest version of which (http://www.atgc-montpellier.fr/phyml-sms/) includes automatic selection of the best-fit substitution model for a given alignment. The best model identified by PhyML was LG +G6 +I +F. LG, Le-Gascuel matrix; G6 +I +F, Gamma shape parameter: fixed; number of categories: 6; Proportion of Invariable sites: fixed; Equilibrium frequencies: empirical. Branch support values were calculated from 100 independent runs. Maximum likelihood trees for RpoB and GyrB were constructed with MEGA6 (Tamura *et al.*, 2013).

3. Whole-genome-based trees

For genome-based trees, we performed BLAST comparisons between each pair of genomes (DNA assemblies), using MegaBLAST (BLASTN 2.3.1.+, Morgulis *et al.*, 2008) run with default parameters, except for several minor changes specified in the legend to Figure S3A and that the high-scoring segments (HSPs) in the output were filtered to prevent multiple hits from the same DNA segment. BLAST results were used to determine the alignment fraction (AF), the percentage of conserved DNA shared by the two genomes, following the principles described by Goris *et al*. (2007), Deloger *et al*. (2009), and Varghese *et al*. (2015). For distantly related organisms (members of different genera), we calculated on the fraction of each genome that contributed to the pairwise alignment, as proposed by Deloger *et al.* (2009). Briefly, the MegaBLAST-reported fraction of the aligned DNA was compared to the total length of genomic DNA for each strain; these values were averaged for each genome pair, converted to distance measures, and used to construct a neighbor-joining tree. These values were also plotted as a two-dimensional display of the results obtained by multi-dimensional scaling (Brover *et al*., manuscript in preparation).

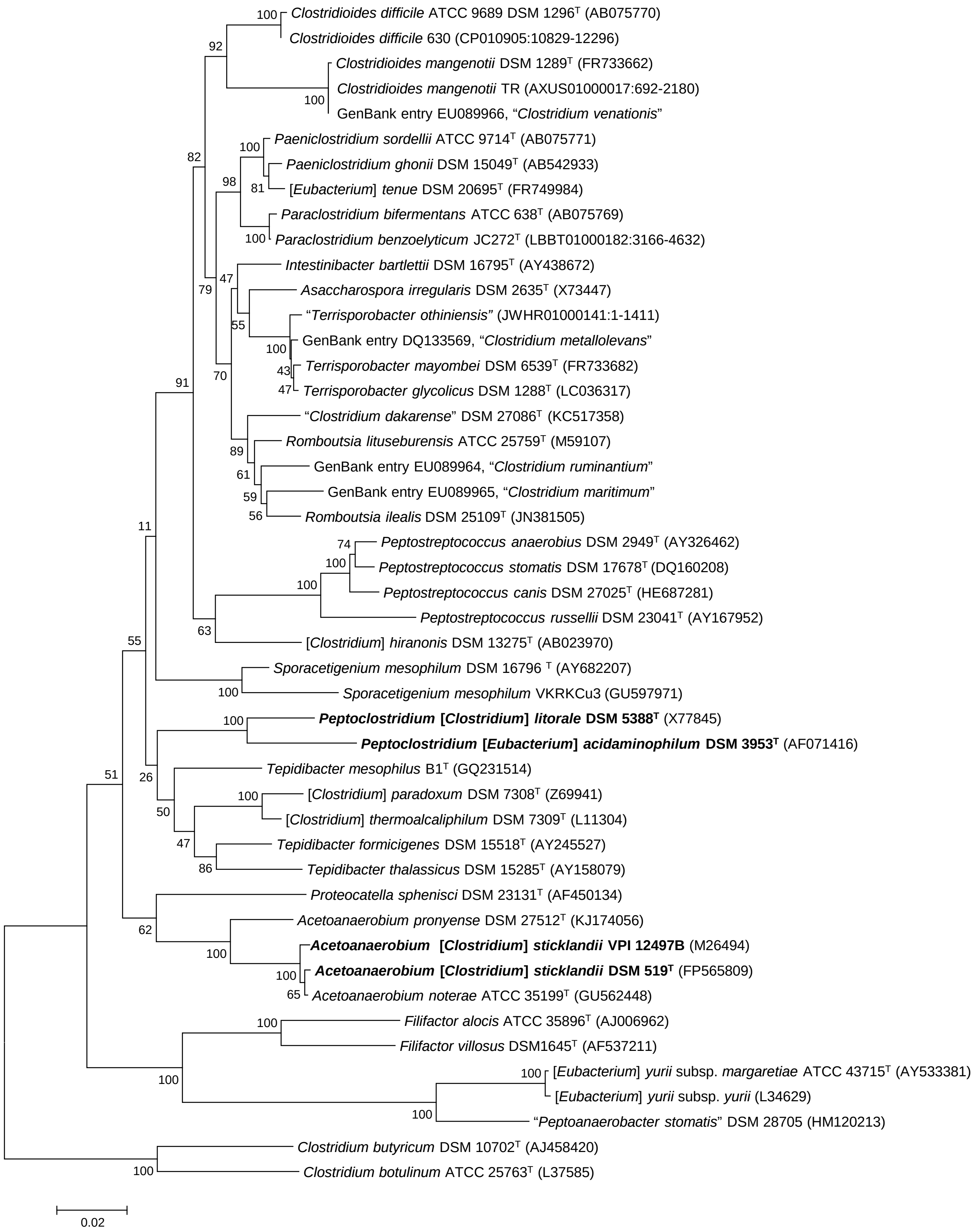

Figure S1. **A neighbor-joining tree of 16S rRNA genes from *Peptostreptococcaceae* members.** The tree was built using MEGA6 and rooted with sequences from *C. botulinum* and *C. butyricum*. GenBank accession numbers for 16S rRNA gene sequences are listed in parentheses. The names of the organisms reclassified in this work are shown in bold. The names of organisms that have not been validly published are in quotation marks, sequences from organisms that have not been deposited in culture collections are listed under their GenBank entries.

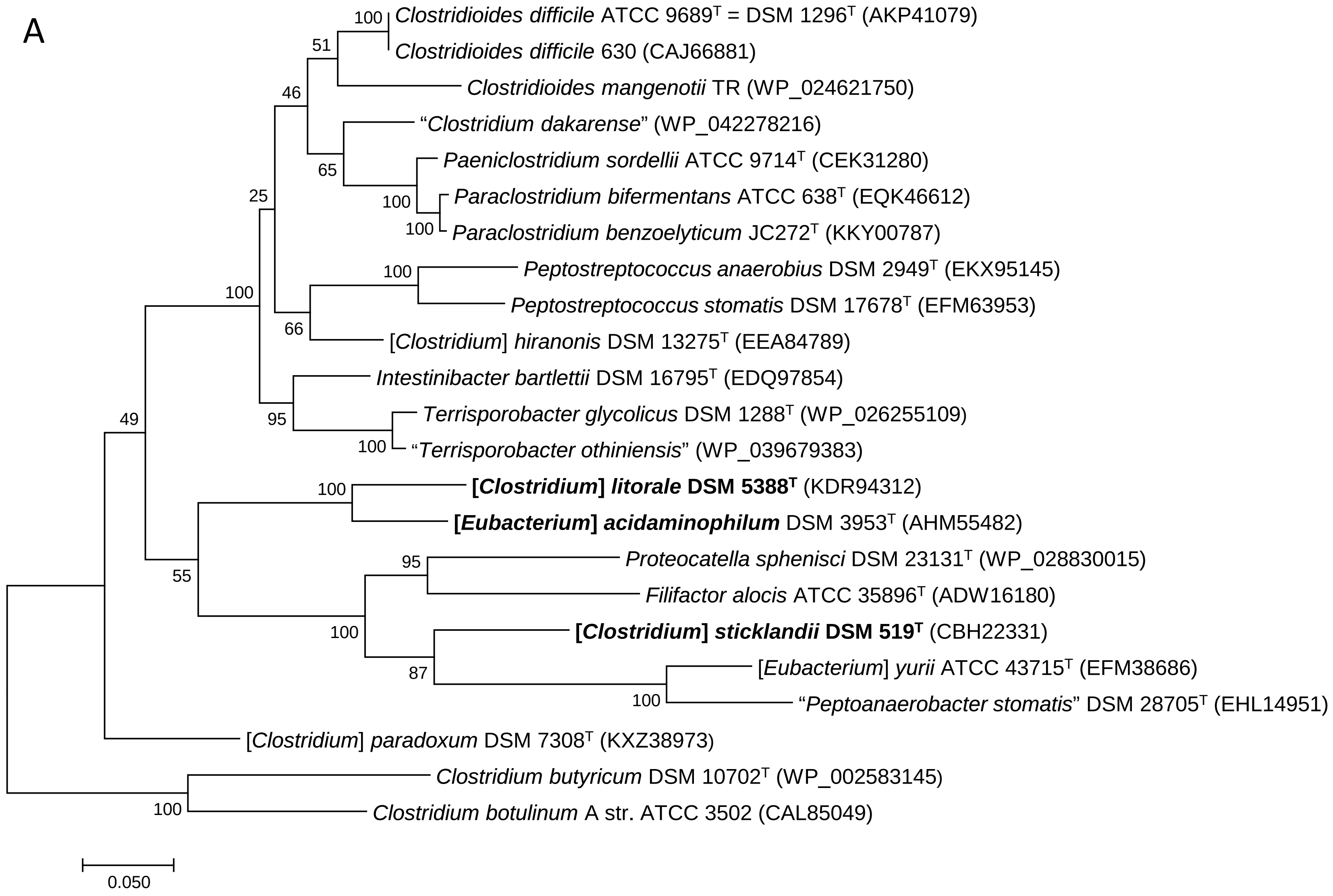

Figure S2. Maximum likelihood trees for DNA-directed RNA polymerase beta subunit (RpoB, panel A, 1182 positions) and DNA gyrase subunit B (GyrB, panel B, 626 positions). The sequences were taken from GenBank (where available, CDS id's are in parentheses) or from NCBI's RefSeq, aligned with MUSCLE and ML trees were constructed with MEGA6 by applying Neighbor-Joining and BioNJ algorithms to a matrix of pairwise distances estimated using a JTT-based model. Protein accession numbers are shown in parentheses. Organisms proposed for renaming are in bold, quotation marks indicate the species that had not been validly described at the time of this writing.

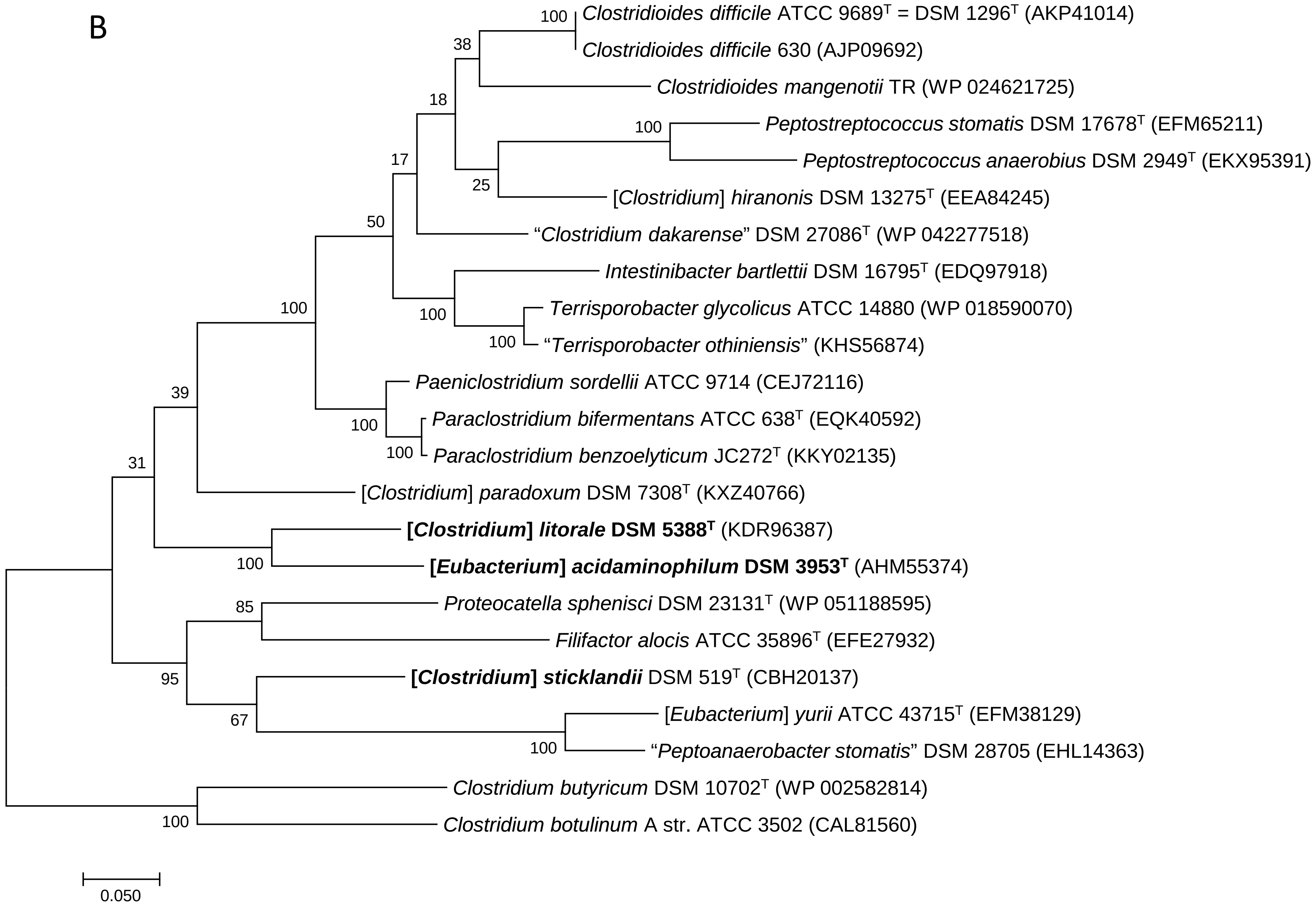
B
Clostridioides difficile ATCC 9689^{T} = DSM 1296^{T} (AKP41014)
Clostridioides difficile 630 (AJP09692)
Clostridioides mangenotii TR (WP 024621725)
Peptostreptococcus stomatis DSM 17678^{T} (EFM65211)
Peptostreptococcus anaerobius DSM 2949^{T} (EKX95391)
[Clostridium] hiranonis DSM 13275^{T} (EEA84245)
"Clostridium dakarense" DSM 27086^{T} (WP 042277518)
Intestinibacter bartlettii DSM 16795^{T} (EDQ97918)
Terrisporobacter glycolicus ATCC 14880 (WP 018590070)
"Terrisporobacter othiniensis" (KHS56874)
Paeniclostridium sordellii ATCC 9714 (CEJ72116)
Paraclostridium bifermentans ATCC 638^{T} (EQK40592)
Paraclostridium benzoelyticum JC272^{T} (KKY02135)
[Clostridium] paradoxum DSM 7308^{T} (KXZ40766)
[Clostridium] litorale DSM 5388^{T} (KDR96387)
[Eubacterium] acidaminophilum DSM 3953^{T} (AHM55374)
Proteocatella sphenisci DSM 23131^{T} (WP 051188595)
Filifactor alocis ATCC 35896^{T} (EFE27932)
[Clostridium] sticklandii DSM 519^{T} (CBH20137)
[Eubacterium] yurii ATCC 43715^{T} (EFM38129)
"Peptoanaerobacter stomatis" DSM 28705 (EHL14363)
Clostridium butyricum DSM 10702^{T} (WP 002582814)
Clostridium botulinum A str. ATCC 3502 (CAL81560)
100
38
18
100
25
17
50
100
100
100
39
100
100
31
100
85
95
67
100
100
0.050

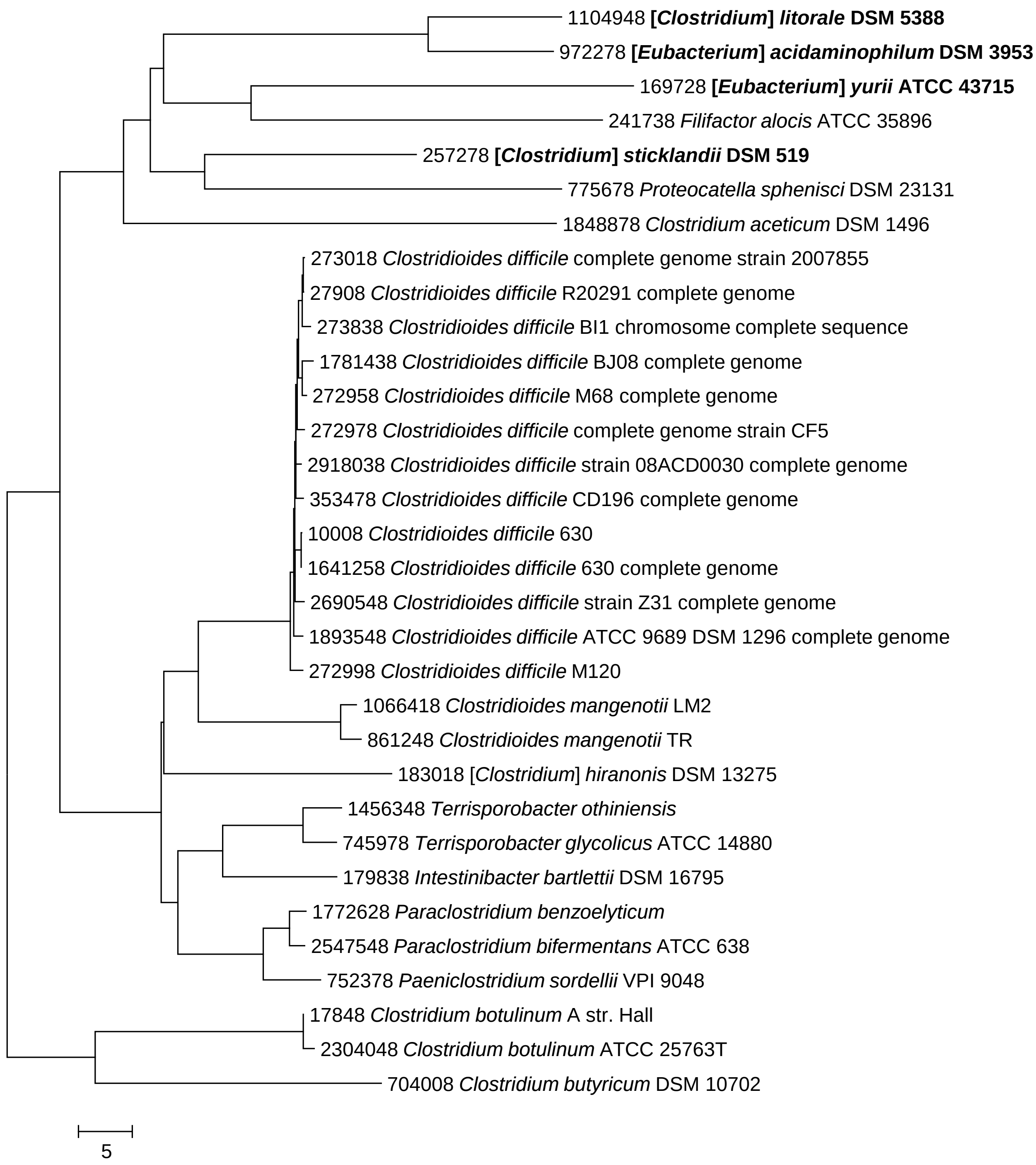

Figure S3. **A tree based on whole-genome alignments (A) and its visualization using principal components analysis (B).** The tree is based on the "conservation" scores, derived from the BLAST scores of pairwise genome alignments and rooted with genomes of *Clostridium botulinum* and *C. butyricum*. The numbers in front of the organism names indicate genomic entries in the NCBI Assembly database, e.g. 1104948 for *C. litorale* indicates www.ncbi.nlm.nih.gov/assembly/1104948.

Pairwise genome alignments were obtained using MegaBLAST with the following parameters: -max_target_seqs 100000; -xdrop_gap 150; -penalty -1; -gapopen 3; -gapextend 1; -dbsize 10000000; -searchsp 10000000. Aligned genome segments of < 60 bp have been ignored; aligned segments separated by < 400 bp have been merged to form "conserved segments". For each genome, the "conservation" value C has been calculated as the total length of the conserved segments divided by the total length of all available DNA in a genome assembly. The distance d_{cons} between genomes 1 and 2 was calculated as $d_{cons} = -10 * (\log C1 + \log C2)$ and used to build a tree using the neighbor-joining algorithm implemented in the NCBI Genome Workbench http://www.ncbi.nlm.nih.gov/tools/gbench/.

Peptostreptococcaceae

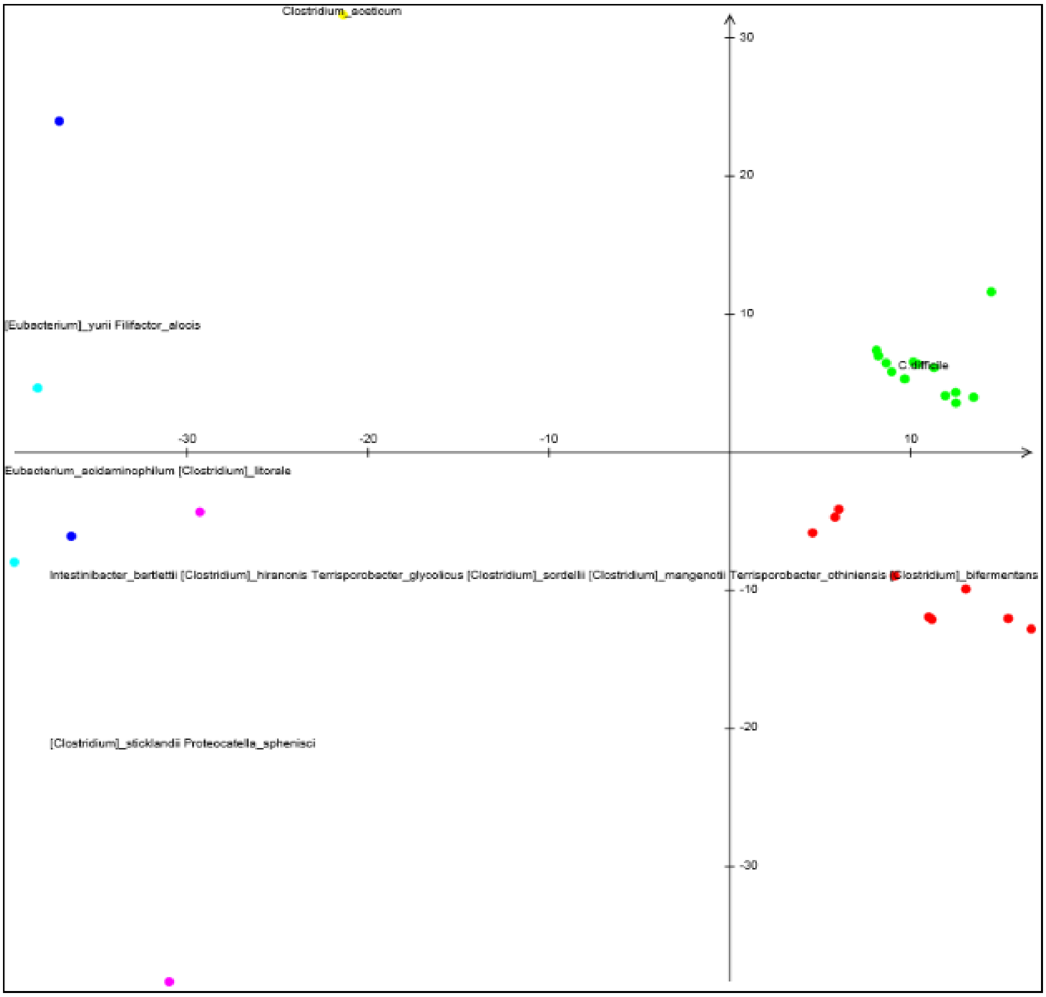

Scale: dist(x,y)/10 ≈ - log(%symmetric_identity/100)
Points: 29

Show names: ☐

Principal Components

Axis	% Explained Distances	% Total
1	37	37
2	16	53
3	15	68
4	10	78

Clusters

Cluster	# Points	Species
1	2	[Eubacterium]_yurii(1) Filifactor_alocis(1)
2	13	C.difficile
3	2	Eubacterium_acidaminophilum(1) [Clostridium]_litorale(1)
4	9	Intestinibacter_bartlettii(1) [Clostridium]_hiranonis(1) Terrisporobacter_glycolicus(1) [Clostridium]_sordellii(1) [Clostridium] _mangenotii(2) Terrisporobacter_othiniensis(1) [Clostridium]_bifermentans(1)
5	2	[Clostridium]_sticklandii(1) Proteocatella_sphenisci(1)
6	1	Clostridium_aceticum

Figure S3B. Pairwise distances obtained from whole-genome alignments visualized using principal component analysis. The dots in cyan correspond to the cluster formed by *C. litorale* and *E. acidaminophlum*.

Table S1. Genome sequences of the members of the *Peptostreptococcaceae*[a]

Organism name[b]	Genome size, kb	Pro-teins	%GC	Genome ID	GenBank accession, reference
**Complete genomes**					
Clostridioides difficile DSM 1296[T]	4,213	3,782	28.8	535	CP011968 (Riedel *et al.*, 2015)
Clostridioides difficile 630	4,298	3,767	29.1	535	AM180355 (Sebaihia *et al.*, 2006; Monot *et al.*, 2011)
[*Clostridium*] *sticklandii* DSM 519[T]	2,715	2,476	33.3	1723	FP565809 (Fonknechten *et al.*, 2010)
[*Eubacterium*] *acidaminophilum* DSM 3953[T]	3,055	2,789	44.0	31356	CP007452 (Poehlein *et al.*, 2014b)
Filifactor alocis ATCC 35896[T]	1,931	1,644	35.4	2011	CP002390
**Draft genomes**					
[*Clostridium*] *hiranonis* DSM 13275[T]	2,480	2,123	31.0	2045	ABWP00000000
[*Clostridium*] *litorale* DSM 5388[T]	3,405	3,002	41.3	32065	JJMM00000000 (Poehlein *et al.*, 2014a)
[*Clostridium*] *mangenotii* TR	3,024	2,699	31.3	24505	AXUS00000000 (McLaughlin *et al.*, 2014)
[*Clostridium*] *paradoxum* DSM 7308[T]	1,926	1,937	30.0	44033	LSFY00000000 (Lancaster *et al.*, 2016)
[*Eubacterium*] *yurii* ATCC 43715[T]	2,511	2,014	32.6	2999	AEES00000000
Intestinibacter bartlettii DSM 16795[T]	2,961	2,583	28.8	961	ABEZ00000000
Paeniclostridium sordellii ATCC 9714[T]	3,553	3,271	27.4	13400	APWR00000000 (Sirigi Reddy *et al.*, 2013)
Paeniclostridium sordellii VPI 9048[c]	3,572	3,985	27.3	13400	AQGJ00000000 (Sirigi Reddy *et al.*, 2013)
Paraclostridium benzoelyticum JC272[T]	3,556	3,242	32.2	–	LBBT00000000 (Tushar *et al.*, 2015; Sasi Jyothsna *et al.*, 2016)
Paraclostridium bifermentans ATCC 638[T]	3,604	3,369	28.4	15093	AVNC00000000
Paraclostridium bifermentans WYM	3,476	3,225	28.0	15093	AVSU00000000 (Wong *et al.*, 2014)
Peptostreptococcus anaerobius DSM 2949[T]	2,107	1,904	35.7	1958	ARMA00000000
Proteocatella sphenisci DSM 23131[T]	2,411	2,198	36.5	30045	AUID00000000
Terrisporobacter glycolicus ATCC 14880[T]	4,020	3,746	28.4	14311	AUUB00000000
"Clostridium dakarense" DSM 27086[T]	3,736	3,344	28.0	40074	CBTZ000000000 (Lo *et al.*, 2013)
"Peptoanaerobacter stomatis" DSM 28705[T] (=Peptostreptococcaceae bacterium ACC19a)	2,544	2,050	30.3	2952	AFZE00000000 (Sizova *et al.*, 2015)
"Terrisporobacter othiniensis" 08-306576	3,987	3,634	28.5	36713	JWHR00000000 (Lund *et al.*, 2015)
Clostridium sensu stricto					
Clostridium butyricum DSM 10702[T]	4,597	3.976	28.5	1593	AQQF00000000 (Xin *et al.*, 2013)
Clostridium botulinum A str. ATCC 3502	3,903	3,590	28.2	726	AM412317 (Sebaihia *et al.*, 2007)

[a] - As of July 1st, 2016. Only those genomes used in the phylogenetic trees (Fig. 1-2, S1-S3) are listed here.

[b] - No suitable genomes for *Romboutsia* spp. were available at the time. *Romboutsia* sp. MT17 (GenBank: FJTZ00000000) had not been described and its 16S rRNA was only 96% identical to that of the type species *Romboutsia ilealis* DSM 25109[T]. Misnamed *"Clostridium lituseburense"* L74, whose genome was described by Lee *et al.* (2016), is not a member of *Peptostreptococcaceae*.

[c] - Draft genome of *P. sordellii* VPI 9048 only includes partial 16S rRNA sequence, which is 99% identical to that of the type strain *P. sordellii* ATCC 9714[T].

Table S2. Characteristics of the proposed *Peptoclostridium litorale* and *Peptoclostridium acidaminophilum* compared to the type species of the other *Peptostreptococcaceae* genera.

1, *Peptoclostridium litorale* W6 = ATCC 49638^T = DSM 5388^T (Fendrich *et al.*, 1990; Poehlein *et al.*, 2014a); 2, *Peptoclostridium acidaminophilum* al-2 = ATCC 49065^T = DSM 3953^T (Zindel *et al.*, 1988; Poehlein *et al.*, 2014b). Other organisms are listed in the same order as in Figure 1: 3, *Clostridioides difficile* ATCC 9689^T = DSM 1296^T = JCM 1296^T (data from Rainey *et al.*, 2009; Scaria *et al.*, 2015); 4, *Paraclostridium bifermentans* ATCC 638^T = DSM 14991^T = JCM 1386^T (Chamkha *et al.*, 2001b; Sasi Jyothsna *et al.*, 2016); 5, *Paeniclostridium sordellii* ATCC 9714^T = LMG 15708^T = JCM 3814^T (Rainey *et al.*, 2009; Scaria *et al.*, 2015; Sasi Jyothsna *et al.*, 2016); 6, *Romboutsia ilealis* CRIB^T = DSM 25109^T = NIZO 4048^T (Gerritsen *et al.*, 2014); 7, *Asaccharospora irregularis* ATCC 25756^T = DSM 2635^T = JCM 1425^T (Rainey *et al.*, 2009; Gerritsen *et al.*, 2014); 8, *Intestinibacter bartlettii* WAL 16138^T = ATCC BAA-827^T = DSM 16795^T (Song *et al.*, 2004; Gerritsen *et al.*, 2014); 9, *Terrisporobacter glycolicus* ATCC 14880^T = DSM 1288^T = JCM 1401^T (Chamkha *et al.*, 2001a; Gerritsen *et al.*, 2014); 10, *Peptostreptococcus anaerobius* ATCC 27337^T = DSM 2949^T = LMG 15865^T (Ezaki, 2009); 11, *Acetoanaerobium noterae* NOT-3^T = ATCC 35199^T (Sleat *et al.*, 1985; Rainey, 2009; Bes *et al.*, 2015); 12, *Proteocatella sphenisci* ATCC BAA-755^T = DSM 23131^T = JCM 12175^T (Pikuta *et al.*, 2009); 13, *Filifactor villosus* ATCC 33388^T = DSM 1645^T (Love *et al.*, 1979; Jalava & Eerola, 2009) (some data are for *Filifactor alocis*); 14, "*Peptoanaerobacter stomatis*" ACC19a^T = ATCC BAA-2665^T = DSM 28705^T (Sizova *et al.*, 2015); 15, *Sporacetigenium mesophilum* ZLJ115^T = DSM 16796^T (Chen *et al.*, 2006); 16, *Tepidibacter thalassicus* SC 562^T = DSM 15285^T= UNIQEM 215^T (Slobodkin *et al.*, 2003; Slobodkin, 2009; Tan *et al.*, 2012). The ± sign indicates reported weak reaction, empty cells indicate the absence of available data.

**Property**	**Organisms**															
	**1**	**2**	**3**	**4**	**5**	**6**	**7**	**8**	**9**	**10**	**11**	**12**	**13**	**14**	**15**	**16**
**Genome size, Mb**	3.40	3.06	4.21	3.60	3.55			2.96	4.02	2.11		2.41		2.54		
**GC%**	41.3	44.0	28.8	28.4	27.4	28.1	27	29.8	29.1	35.7	36.8	39.5	34	30.3	53.9	24.9
**Cell size, µM**																
Width	1.0-1.5	0.7-1.0	0.5-1.9	0.5-1.9	0.5-1.7	1-2	0.8–1.6	1.0–1.5	0.4-1.1	0.8	0.8	0.7–0.8	0.6	0.4-0.8	0.9–1.0	0.7-0.9
Length	2.0-8.0	1.3-6.0	3.0-17	1.5-14	1.6-20	1-5.3	3.5–12.6	5.0–50	2.0-15	0.8-0.9	1-5	3.0–5.0	4.0-6.0	1.2-2.5	3.6–7.3	3.5-6.0
**Cell shape**	Rods	Rods	Rods	Rods	Rods	Rods	Rods	Rods	Rods	Cocci	Rods	Rods	Rods	Rods	Rods	Rods
**Gram-staining**	–	+	+	+	+	+	+	+	+	+	–	+	±	+	+	+
**Spore formation**	+	–	+	+	+	+	+	±	+	–	–	+	+	–	+	+
**Motility**	+	+	+	+	+	–	+	–	+	–	+	+	–	+	+	+
**Optimal temp., °C**	28	32-36	30-37	30-37	30-37	37	30-37	37	30-37	37	37	29	37	37	37-39	50
**Optimal pH**	7.3	7.1-7.4	6.0	7.0	6.0	7.0-7.5			7.3-7.5		7.6	8.3		7.0	7.5	6.5-6.8
**Hydrolysis of**																
Casein	–			+	+		–			–						+
Gelatin	–	–		+	+	–	+	–	–	+			±	–	–	–
Starch	–		–	–		–			–			+			+	+
**Enzymes**																
Catalase	–	–	–	–	–	–		–		–	–	–			–	
Oxidase	–	–	–	–	–						–				–	
Lecithinase\lipase	–	–	–	+		+		–	–						–	
Urease	–	–		–	–	–		–		–					–	

Property	Organisms															
	1	2	3	4	5	6	7	8	9	10	11	12	13	14	15	16
**Utilization of sugars**																
L-Arabinose	–	–	–	–		–	–		–	–	–		–	–	+	–
D-Cellobiose	–		±	–		–		+	–		–	–		–	±	–
D-Fructose	–	–	+	+	+	–	–	+	+		–	–	–		+	–
D-Galactose	–		–	–		+	–	–	–		–		–		±	–
D-Glucose	–	–	+	+	+	+	–	+	+	±	+	–	–	+	+	+
Lactose	–		–	–	–	–	–	–	–	–	–	–	–	–	–	
Maltose	–	–	–	+	+	–	–	+	+		+	–	–	+	+	+
D-Mannitol	–		+	–	–	–	–	+	–	–	–	–	–	–	–	–
D-Mannose	–	–	+	–	–	–	–	±	–	±	–	–	–	–	±	
D-Ribose	–		–	–	–	–	–	+	–		–	–	–		+	–
D-Sorbitol	–		±	–	–	–	–	+	+	–	–			–	–	–
Sucrose	–	–	–	–	–	+	–	+	–	–	–	–	–	–	±	–
D-Xylose	–		±	–	–	–	–	–	+		–		–	–	+	–
**Reduction of**																
Nitrate	–	–	–	–	–			–	–					–	–	–
Sulfate	–	–				–		–	–							–
Sulfite	–					+		–	–							–
Thiosulfate	–	–				–		–	–							–
**Acetate production**	+	+	+			+	+	+	+	+	+	+	+	+	+	+
**H_2 produced**	–	±	+	±	+	+	±	–	+		–	±			+	
**H_2S produced**	–		+	–											+	+
**NH_3 produced**	+	+	+			+	+						+		+	
**Predominant fatty acids**				$C_{16:0}$	$C_{16:0}$; $C_{18:0}$	$C_{16:0}$; $C_{17:0}$	$C_{16:0}$; $C_{16:1}\omega 7c$	$C_{18:1}$; $C_{16:0}$	$C_{16:0}$			$C_{14:0}$; $C_{16:0}$		$C_{14:0}$	$C_{14:0}$; $C_{16:0}$, $C_{16:1}\omega 7c$	

Table S3. Putative sporulation proteins in *Peptoclostridium litorale* and *P. acidaminophilum*[a]

Gene name (*B. subtilis*)	*Clostridium litorale* CDS	*Eubacterium acidamino-philum* CDS	Iden-tity, %	BLAST E-value
spo0A	KDR94118	AHM56503	49.0	8E-73
spo0J	KDR96372	AHM57500	54.6	3E-71
sigE	KDR95145	AHM56685	85.2	1E-163
sigF	KDR96393	AHM55380	91.1	3E-137
spoIIAA	KDR96391	AHM55378	83.8	2E-48
spoIIAB	KDR96392	AHM55379	76.8	4E-61
spoIID	KDR96081	AHM56263	47.7	4E-137
spoIIE	KDR95541	AHM56178	81.2	1E-199
spoIIQ	KDR94841	AHM55716	63.1	1E-139
spoIIIJ	KDR96378	AHM57505	76.5	2E-97
spoIVFB	KDR93751	AHM56979	67.1	7E-156
spoVB	KDR94240	AHM55549	75.6	1E-199
spoVC	KDR94243	AHM55546	73.0	4E-72
spoVD	KDR95016	AHM56340	69.5	1E-199
spoVE	KDR95021	AHM56345	70.8	2E-149
spoVG	KDR94246	AHM55543	81.4	8E-39
spoVS	KDR95155	AHM56669	94.2	2E-42
abrB	KDR94262	AHM55532	97.5	7E-41
slrR	KDR95600	AHM57621	41.4	2E-35
alr	KDR94822	AHM55700	74.0	2E-180
cwlD	KDR95675	AHM56020	44.0	3E-96
dacB	KDR94090	AHM56475	57.9	2E-123
dapA	KDR95311	AHM56886	81.4	4E-139
dapB	KDR95310	AHM56887	70.1	2E-104
etfA	KDR95723	AHM56121	58.0	7E-160
obgE	KDR94074	AHM56460	76.8	1E-199
stoA	KDR95762	AHM58259	63.0	2E-50
sleL	KDR96768	AHM57802	71.0	1E-199
spsC	KDR94625	AHM57300	36.2	2E-66
spsI	KDR94588	AHM55662	35.8	9E-20
spsJ	KDR94586	AHM57182	26.2	1E-26
spsK	KDR94585	AHM57303	34.8	4E-05
yerB	KDR95388	AHM56066	41.0	3E-51
yhaX	KDR96655	AHM58049	62.7	5E-103
ykvI	KDR96160	AHM58009	62.3	1E-137
yoaR	KDR95550	AHM56169	49.9	3E-127
yocH	KDR94252	AHM55538	57.6	2E-108
yqfU	KDR94469	AHM57128	62.7	5E-104
yqhQ	KDR94799	AHM55677	74.9	2E-118
yviA	KDR95547	AHM56172	63.1	1E-105
yyaC	KDR96146	AHM56310	63.9	3E-69

[a] – The list of known and putative sporulation genes of *Bacillus subtilis* is taken from (Galperin *et al.*, 2012). The table lists the GenBank accession (CDS) numbers of the corresponding proteins from *C. litorale* and *E. acidaminophilum*, their identity levels and expectation values obtained from pairwise BLAST comparisons. Spo proteins are hyperlinked to their GenBank entries.

Table S4. Recently proposed changes in the names of the *Peptostreptococcaceae* members[a]

Organism name[a]	Updated name	Reference
Clostridium bartlettii	*Intestinibacter bartlettii*	Gerritsen *et al.* (2014)
Clostridium bifermentans	*Paraclostridium bifermentans*	Sasi Jyothsna *et al.* (2016)
Clostridium difficile	*Clostridioides difficile*	Lawson *et al.* (2016)
Clostridium ghonii	*Paeniclostridium ghonii*	Sasi Jyothsna *et al.* (2016)
Clostridium glycolicum	*Terrisporobacter glycolicus*	Gerritsen *et al.* (2014)
Clostridium hiranonis	–	–
Clostridium irregulare	*Asaccharospora irregularis*	Gerritsen *et al.* (2014)
Clostridium litorale	*Peptoclostridium litorale*	This work
Clostridium lituseburense	*Romboutsia lituseburensis*	Gerritsen *et al.* (2014)
Clostridium mangenotii	*Clostridioides mangenotii*	Lawson *et al.* (2016)
Clostridium mayombei[a]	*Terrisporobacter mayombei*	Gerritsen *et al.* (2014)
Clostridium paradoxum	–	–
Clostridium sordellii	*Paeniclostridium sordellii*	Sasi Jyothsna *et al.* (2016)
Clostridium sticklandii	*Acetoanaerobium sticklandii*	Bes *et al.* (2015), this work
Clostridium thermoalcaliphilum[a]	–	–
Eubacterium acidaminophilum	*Peptoclostridium acidaminophilum*	This work
Eubacterium tenue	–	–
Eubacterium yurii	–	–

[a] - This table includes 15 validly described species that have been assigned to the family *Peptostrepto-coccaceae* in the 2nd edition of Bergey's (Ludwig *et al.*, 2009) with the addition of two more species, *C. mayombei* and *C. thermoalcaliphilum* (Kane *et al.*, 1991; Li *et al.*, 1994), which had been assigned to Cluster XI by Collins *et al*. (1994), but not assigned to *Peptostreptococcaceae* in Bergey's (Ludwig *et al.*, 2009; Rainey *et al.*, 2009). *Eubacterium acidaminophilum* was not mentioned by either Collins *et al*. (1994) or Ludwig *et al.* (2009). A dash indicates that the organism's name remains to be updated.

Table S5. Updated nomenclature of the family *Peptostreptococcaceae*[a]

Organism name[a]	Comment, reference
Acetoanaerobium	This genus has been assigned to *Clostridiales* Family XIX *Incertae Sedis* in Bergey's (Ludwig *et al.*, 2009; Rainey, 2009) but listed within *Peptostreptococaceae* in the SILVA database (Yilmaz *et al.*, 2014)
Acetoanaerobium noterae	Sleat *et al.* (1985); Rainey (2009)
Acetoanaerobium pronyense	Bes *et al.* (2015)
Acetoanaerobium sticklandii	Recognized as a close relative of *A. pronyense* by Bes *et al.* (2015), reclassification proposed in in this work
Asaccharospora	This genus was first proposed in Gerritsen *et al.* (2014)
Asaccharospora irregularis	Gerritsen *et al.* (2014)
Clostridioides	This genus was first proposed in Lawson *et al.* (2016)
Clostridioides difficile	Lawson *et al.* (2016)
Clostridioides mangenotii	Lawson *et al.* (2016)
Filifactor	Jalava & Eerola (2009)
Filifactor alocis	Jalava & Eerola (2009)
Filifactor villosus	Jalava & Eerola (2009)
***Intestinibacte*r**	This genus was first proposed in Gerritsen *et al.* (2014)
Intestinibacter bartlettii	Gerritsen *et al.* (2014)
Paeniclostridium	This genus was first proposed in Sasi Jyothsna *et al.* (2016)
Paeniclostridium ghonii	Sasi Jyothsna *et al.* (2016)
Paeniclostridium sordelii	Sasi Jyothsna *et al.* (2016)
Paraclostridium	This genus was first proposed in Sasi Jyothsna *et al.* (2016)
Paraclostridium benzoelyticum	Sasi Jyothsna *et al.* (2016)
Paraclostridium bifermentans	Sasi Jyothsna *et al.* (2016)
Peptoanaerobacter	Described as a new genus in Sizova *et al.* (2015), not recognized as validly described at the time of this writing
Peptoanaerobacter stomatis	Sizova *et al.* (2015)
Peptoclostridium	This genus was first proposed in Yutin & Galperin (2013), the proposal modified in this work
Peptoclostridium acidaminophilum	Zindel *et al.* (1988); this work
Peptoclostridium litorale	Fendrich *et al.* (1990); Rainey *et al.* (2009); this work
Peptostreptococcus	Ezaki (2009)
Peptostreptococcus anaerobius	Paster *et al.* (1993); Ezaki (2009)
Peptostreptococcus canis	Lawson *et al.* (2012)
Peptostreptococcus russellii	Whitehead *et al.* (2011)
Peptostreptococcus stomatis	Downes & Wade (2006)

Proteocatella	Described as a new genus in Cluster XI by Pikuta *et al.* (2009); listed in *Peptostreptococcaceae* in SILVA but in 'unclassified Clostridiales' in the NCBI Taxonomy database
Proteocatella sphenisci	Pikuta *et al.* (2009)
Romboutsia	This genus was first proposed in Gerritsen *et al.* (2014)
Romboutsia ilealis	Gerritsen *et al.* (2014)
Romboutsia lituseburensis	Gerritsen *et al.* (2014)
Romboutsia sedimentorum	Wang *et al.* (2015)
Sporacetigenium	Chen *et al.* (2006)
Sporacetigenium mesophilum	Chen *et al.* (2006)
Tepidibacter	Slobodkin *et al.* (2003); Slobodkin (2009); Tan *et al.* (2012)
Tepidibacter formicigenes	Urios *et al.* (2004); Slobodkin (2009)
Tepidibacter mesophilus	Tan *et al.* (2012)
Tepidibacter thalassicus	Slobodkin *et al.* (2003); Slobodkin (2009)
Terrisporobacter	This genus was first proposed in Gerritsen *et al.* (2014)
Terrisporobacter glycolicus	Gerritsen *et al.* (2014)
Terrisporobacter mayombei	Gerritsen *et al.* (2014)
"*Terrisporobacter othiniensis*"	Lund *et al.* (2015)
Terrisporobacter petrolearius	Deng *et al.* (2015)
**Unassigned [*Clostridium*] spp.**	
[*Clostridium*] *hiranonis*	Kitahara *et al.* (2001); Rainey *et al.* (2009)
[*Clostridium*] *paradoxum*	Li *et al.* (1993); Rainey *et al.* (2009)
[*Clostridium*] *thermoalcaliphilum*	Li *et al.* (1994); Rainey *et al.* (2009)
**Unassigned [*Eubacterium*] spp.**	
Eubacterium tenue	Recognized as a close relative of *Paeniclostridium ghonii* by Gerritsen *et al.* (2014) and Sasi Jyothsna *et al.* (2016), could be reclassified as *Paeniclostridium tenue*
Eubacterium yurii	Recognized as a close relative of *Peptoanaerobacter stomatis* by Sizova *et al.* (2015), could be reclassified as *Peptoanaerobacter yurii*
**Organisms without valid description**	
"*Clostridium dakarense*"	Deposited in DSM (DSM 27086) and CSUR (CSUR P243), can be reassigned as *Romboutsia dakarense* (Lo *et al.*, 2013)
"Clostridium maritimum"	*Romboutsia sp.*, not deposited in any culture collection
"Clostridium metallolevans"	*Terrisporobacter sp.*, not deposited in any culture collection
"Clostridium ruminantium"	*Romboutsia sp.*, not deposited in any culture collection
"*Clostridium venationis*"	*Clostridioides mangenotii*, not deposited in any collection

[a] This listing takes into account the classification presented in the 2nd edition of Bergey's (Ludwig *et al.*, 2009) and subsequent proposals and assignments, see Figure 1 and the entries in SIlVA database https://www.arb-silva.de/browser/ssu-126/AB075770 (Quast *et al.*, 2013; Yilmaz *et al.*, 2014) and the NCBI Taxonomy Database http://www.ncbi.nlm.nih.gov/Taxonomy/Browser/wwwtax.cgi? id=186804 (Federhen, 2012, 2015).

References

Benson, D. A., Clark, K., Karsch-Mizrachi, I., Lipman, D. J., Ostell, J. & Sayers, E. W. (2015). GenBank. *Nucleic Acids Res* **43**: D30-D35.

Bes, M., Merrouch, M., Joseph, M., Quemeneur, M., Payri, C., Pelletier, B., Ollivier, B., Fardeau, M. L., Erauso, G. & other authors (2015). *Acetoanaerobium pronyense* sp. nov., an anaerobic alkaliphilic bacterium isolated from a carbonate chimney of the Prony Hydrothermal Field (New Caledonia). *Int J Syst Evol Microbiol* **65**: 2574-2580.

Chamkha, M., Labat, M., Patel, B. K. & Garcia, J. L. (2001a). Isolation of a cinnamic acid-metabolizing *Clostridium glycolicum* strain from oil mill wastewaters and emendation of the species description. *Int J Syst Evol Microbiol* **51**: 2049-2054.

Chamkha, M., Patel, B. K. C., Garcia, J. L. & Labat, M. (2001b). Isolation of *Clostridium bifermentans* from oil mill wastewaters converting cinnamic acid to 3-phenylpropionic acid and emendation of the species. *Anaerobe* **7**: 189-197.

Chen, S., Song, L. & Dong, X. (2006). *Sporacetigenium mesophilum* gen. nov., sp. nov., isolated from an anaerobic digester treating municipal solid waste and sewage. *Int J Syst Evol Microbiol* **56**: 721-725.

Collins, M. D., Lawson, P. A., Willems, A., Cordoba, J. J., Fernandez-Garayzabal, J., Garcia, P., Cai, J., Hippe, H. & Farrow, J. A. E. (1994). The phylogeny of the genus *Clostridium*: proposal of five new genera and eleven new species combinations. *Int J Syst Bacteriol* **44**: 812-826.

Deloger, M., El Karoui, M. & Petit, M. A. (2009). A genomic distance based on MUM indicates discontinuity between most bacterial species and genera. *J Bacteriol* **191**: 91-99.

Deng, Y., Guo, X., Wang, Y., He, M., Ma, K., Wang, H., Chen, X., Kong, D., Yang, Z. & other authors (2015). *Terrisporobacter petrolearius* sp. nov., isolated from an oilfield petroleum reservoir. *Int J Syst Evol Microbiol* **65**: 3522-3526.

Downes, J. & Wade, W. G. (2006). *Peptostreptococcus stomatis* sp. nov., isolated from the human oral cavity. *Int J Syst Evol Microbiol* **56**: 751-754.

Edgar, R. C. (2004). MUSCLE: multiple sequence alignment with high accuracy and high throughput. *Nucleic Acids Res* **32**: 1792-1797.

Ezaki, T. (2009) Genus I. *Peptostreptococcus*. In *Bergey's Manual of Systematic Bacteriology, 2nd edn, vol 3: The Firmicutes*, pp. 1008-1009. Edited by P. De Vos, G. M. Garrity, D. Jones, N. R. Krieg, W. Ludwig, F. A. Rainey, K.-H. Schleifer & W. B. Whitman. New York: Springer.

Federhen, S. (2012). The NCBI Taxonomy database. *Nucleic Acids Res* **40**: D136-D143.

Federhen, S. (2015). Type material in the NCBI Taxonomy Database. *Nucleic Acids Res* **43**: D1086-D1098.

Fendrich, C., Hippe, H. & Gottschalk, G. (1990). *Clostridium halophilium* sp. nov. and *C. litorale* sp. nov., an obligate halophilic and a marine species degrading betaine in the Stickland reaction *Arch Microbiol* **154**: 127-132.

Fonknechten, N., Chaussonnerie, S., Tricot, S., Lajus, A., Andreesen, J. R., Perchat, N., Pelletier, E., Gouyvenoux, M., Barbe, V. & other authors (2010). *Clostridium sticklandii*, a specialist in amino acid degradation:revisiting its metabolism through its genome sequence. *BMC Genomics* **11**: 555.

Galperin, M. Y., Mekhedov, S. L., Puigbo, P., Smirnov, S., Wolf, Y. I. & Rigden, D. J. (2012). Genomic determinants of sporulation in *Bacilli* and *Clostridia*: towards the minimal set of sporulation-specific genes. *Environ Microbiol* **14**: 2870-2890.

Gerritsen, J., Fuentes, S., Grievink, W., van Niftrik, L., Tindall, B. J., Timmerman, H. M., Rijkers, G. T. & Smidt, H. (2014). Characterization of *Romboutsia ilealis* gen. nov., sp. nov., isolated from the gastro-intestinal tract of a rat, and proposal for the reclassification of five closely related members of the genus *Clostridium* into the genera *Romboutsia* gen. nov., *Intestinibacter* gen. nov., *Terrisporobacter* gen. nov. and *Asaccharospora* gen. nov. *Int J Syst Evol Microbiol* **64**: 1600-1616.

Goris, J., Konstantinidis, K. T., Klappenbach, J. A., Coenye, T., Vandamme, P. & Tiedje, J. M. (2007). DNA-DNA hybridization values and their relationship to whole-genome sequence similarities. *Int J Syst Evol Microbiol* **57**: 81-91.

Guindon, S., Dufayard, J. F., Lefort, V., Anisimova, M., Hordijk, W. & Gascuel, O. (2010). New algorithms and methods to estimate maximum-likelihood phylogenies: assessing the performance of PhyML 3.0. *Syst Biol* **59**: 307-321.

Jalava, J. & Eerola, E. (2009) Genus II. *Filifactor*. In *Bergey's Manual of Systematic Bacteriology, 2nd edn, vol 3: The Firmicutes*, pp. 1009-1013. Edited by P. De Vos, G. M. Garrity, D. Jones, N. R. Krieg, W. Ludwig, F. A. Rainey, K.-H. Schleifer & W. B. Whitman. New York: Springer.

Kane, M. D., Brauman, A. & Breznak, J. A. (1991). *Clostridium mayombei* sp. nov., an H_2/CO_2 acetogenic bacterium from the gut of the African soil-feeding termite, *Cubitermes speciosus*. *Arch Microbiol* **156**: 99-104.

Kitahara, M., Takamine, F., Imamura, T. & Benno, Y. (2001). *Clostridium hiranonis* sp. nov., a human intestinal bacterium with bile acid 7alpha-dehydroxylating activity. *Int J Syst Evol Microbiol* **51**: 39-44.

Lancaster, W. A., Utturkar, S. M., Poole, F. L., Klingeman, D. M., Elias, D. A., Adams, M. W. & Brown, S. D. (2016). Near-complete genome sequence of *Clostridium paradoxum* strain JW-YL-7. *Genome Announc* **4**: e00229-00216.

Lawson, P. A., Johnson, C. N., Bengtsson, L., Charalampakis, G., Dahlen, G., Moore, E. & Falsen, E. (2012). *Peptostreptococcus canis* sp. nov., isolated from subgingival plaque from canine oral cavity. *Anaerobe* **18**: 597-601.

Lawson, P. A., Citron, D. M., Tyrrell, K. L. & Finegold, S. M. (2016). Reclassification of *Clostridium difficile* as *Clostridioides difficile* (Hall and O'Toole 1935) Prevot 1938. *Anaerobe* **40**: 95-99.

Lee, Y., Lim, S., Rhee, M. S., Chang, D. H. & Kim, B. C. (2016). Whole-genome sequence of *Clostridium lituseburense* L74, isolated from the larval gut of the rhinoceros beetle, *Trypoxylus dichotomus*. *Genom Data* **7**: 301-302.

Li, Y., Mandelco, L. & Wiegel, J. (1993). Isolation and characterization of a moderately thermophilic anaerobic alkaliphile, *Clostridium paradoxum* sp. nov. *Int J Syst Bacteriol* **43**: 450-460.

Li, Y., Engle, M., Weiss, N., Mandelco, L. & Wiegel, J. (1994). *Clostridium thermoalcaliphilum* sp. nov., an anaerobic and thermotolerant facultative alkaliphile. *Int J Syst Bacteriol* **44**: 111-118.

Lo, C. I., Mishra, A. K., Padhmanabhan, R., Samb, B., Sow, A. G., Robert, C., Couderc, C., Faye, N., Raoult, D. & other authors (2013). Non-contiguous finished genome sequence and description of *Clostridium dakarense* sp. nov. *Stand Genomic Sci* **9**: 14-27.

Love, D. N., Jones, R. F. & Bailey, M. (1979). *Clostridium villosum* sp. nov. from subcutaneous abscesses in cats. *Int J Syst Bacteriol* **29**: 241-244.

Ludwig, W., Schleifer, K.-H. & Whitman, W. B. (2009) Taxonomic outline of the phylum Firmicutes. In *Bergey's Manual of Systematic Bacteriology, 2nd ed, Vol 3: The Firmicutes*, pp. 15-17. Edited by P.

De Vos, G. M. Garrity, D. Jones, N. R. Krieg, W. Ludwig, F. A. Rainey, K.-H. Schleifer & W. B. Whitman. New York: Springer.

Lund, L. C., Sydenham, T. V., Hogh, S. V., Skov, M., Kemp, M. & Justesen, U. S. (2015). Draft genome sequence of "*Terrisporobacter othiniensis*" isolated from a blood culture from a human patient. *Genome Announc* **3**: e00042-00015.

McLaughlin, R. W., Cochran, P. A., Dowd, S. E., Andersen, K., Anderson, N., Brennan, R., Brook, N., Callaway, T., Diamante, K. & other authors (2014). Draft genome sequence of *Clostridium mangenotii* TR, isolated from the fecal material of a timber rattlesnake. *Genome Announc* **2**: e01107-01113.

Monot, M., Boursaux-Eude, C., Thibonnier, M., Vallenet, D., Moszer, I., Medigue, C., Martin-Verstraete, I. & Dupuy, B. (2011). Reannotation of the genome sequence of *Clostridium difficile* strain 630. *J Med Microbiol* **60**: 1193-1199.

Morgulis, A., Coulouris, G., Raytselis, Y., Madden, T. L., Agarwala, R. & Schäffer, A. A. (2008). Database indexing for production MegaBLAST searches. *Bioinformatics* **24**: 1757-1764.

Parte, A. C. (2014). LPSN--list of prokaryotic names with standing in nomenclature. *Nucleic Acids Res* **42**: D613-D616.

Paster, B. J., Russell, J. B., Yang, C. M., Chow, J. M., Woese, C. R. & Tanner, R. (1993). Phylogeny of the ammonia-producing ruminal bacteria *Peptostreptococcus anaerobius*, *Clostridium sticklandii*, and *Clostridium aminophilum* sp. nov. *Int J Syst Bacteriol* **43**: 107-110.

Pikuta, E. V., Hoover, R. B., Marsic, D., Whitman, W. B., Lupa, B., Tang, J. & Krader, P. (2009). *Proteocatella sphenisci* gen. nov., sp. nov., a psychrotolerant, spore-forming anaerobe isolated from penguin guano. *Int J Syst Evol Microbiol* **59**: 2302-2307.

Poehlein, A., Alghaithi, H. S., Chandran, L., Chibani, C. M., Davydova, E., Dhamotharan, K., Ge, W., Gutierrez-Gutierrez, D. A., Jagirdar, A. & other authors (2014a). First insights into the genome of the amino acid-metabolizing bacterium *Clostridium litorale* DSM 5388. *Genome Announc* **2**: e00754-00714.

Poehlein, A., Andreesen, J. R. & Daniel, R. (2014b). Complete genome sequence of amino acid-utilizing *Eubacterium acidaminophilum* al-2 (DSM 3953). *Genome Announc* **2**: e00573-00514.

Quast, C., Pruesse, E., Yilmaz, P., Gerken, J., Schweer, T., Yarza, P., Peplies, J. & Glockner, F. O. (2013). The SILVA ribosomal RNA gene database project: improved data processing and web-based tools. *Nucleic Acids Res* **41**: D590-D596.

Rainey, F. A. (2009) Genus I. *Acetoanaerobium*. In *Bergey's Manual of Systematic Bacteriology, 2nd edn, vol 3: The Firmicutes*, pp. 1190-1191. Edited by P. De Vos, G. M. Garrity, D. Jones, N. R. Krieg, W. Ludwig, F. A. Rainey, K.-H. Schleifer & W. B. Whitman. New York: Springer.

Rainey, F. A., Hollen, B. J. & Small, A. (2009) Genus I. *Clostridium*. In *Bergey's Manual of Systematic Bacteriology, 2nd ed, Volume 3: The Firmicutes*, pp. 738-828. Edited by P. De Vos, G. M. Garrity, D. Jones, N. R. Krieg, W. Ludwig, F. A. Rainey, K.-H. Schleifer & W. B. Whitman. New York: Springer.

Riedel, T., Bunk, B., Wittmann, J., Thurmer, A., Sproer, C., Gronow, S., Liesegang, H., Daniel, R. & Overmann, J. (2015). Complete genome sequence of the *Clostridium difficile* type strain DSM 1296^{T}. *Genome Announc* **3**: e01186-01115.

Sasi Jyothsna, T. S., Tushar, L., Sasikala, C. & Ramana, C. V. (2016). *Paraclostridium benzoelyticum* gen. nov. sp. nov., isolated from marine sediment and reclassification of *Clostridium bifermentans* as

Paraclostridium bifermentans comb. nov. Proposal of a new genus *Paeniclostridium* gen. nov. to accommodate *Clostridium sordellii* and *Clostridium ghonii*. *Int J Syst Evol Microbiol* **66**: 1268-1274.

Scaria, J., Suzuki, H., Ptak, C. P., Chen, J. W., Zhu, Y., Guo, X. K. & Chang, Y. F. (2015). Comparative genomic and phenomic analysis of *Clostridium difficile* and *Clostridium sordellii,* two related pathogens with differing host tissue preference. *BMC Genomics* **16**: 448.

Sebaihia, M., Wren, B. W., Mullany, P., Fairweather, N. F., Minton, N., Stabler, R., Thomson, N. R., Roberts, A. P., Cerdeno-Tarraga, A. M. & other authors (2006). The multidrug-resistant human pathogen *Clostridium difficile* has a highly mobile, mosaic genome. *Nat Genet* **38**: 779-786.

Sebaihia, M., Peck, M. W., Minton, N. P., Thomson, N. R., Holden, M. T., Mitchell, W. J., Carter, A. T., Bentley, S. D., Mason, D. R. & other authors (2007). Genome sequence of a proteolytic (Group I) *Clostridium botulinum* strain Hall A and comparative analysis of the clostridial genomes. *Genome Res* **17**: 1082-1092.

Sirigi Reddy, A. R., Girinathan, B. P., Zapotocny, R. & Govind, R. (2013). Identification and characterization of *Clostridium sordellii* toxin gene regulator. *J Bacteriol* **195**: 4246-4254.

Sizova, M. V., Chilaka, A., Earl, A. M., Doerfert, S. N., Muller, P. A., Torralba, M., McCorrison, J. M., Durkin, A. S., Nelson, K. E. & other authors (2015). High-quality draft genome sequences of five anaerobic oral bacteria and description of *Peptoanaerobacter stomatis* gen. nov., sp. nov., a new member of the family *Peptostreptococcaceae*. *Stand Genomic Sci* **10**: 37.

Sleat, R., Mah, R. A. & Robinson, R. (1985). *Acetoanaerobium noterae* gen. nov., sp. nov.: an anaerobic bacterium that forms acetate from H_2 and CO_2. *Int J Syst Bacteriol* **35**: 10-15.

Slobodkin, A. I., Tourova, T. P., Kostrikina, N. A., Chernyh, N. A., Bonch-Osmolovskaya, E. A., Jeanthon, C. & Jones, B. E. (2003). *Tepidibacter thalassicus* gen. nov., sp. nov., a novel moderately thermophilic, anaerobic, fermentative bacterium from a deep-sea hydrothermal vent. *Int J Syst Evol Microbiol* **53**: 1131-1134.

Slobodkin, A. I. (2009) Genus III. *Tepidibacter*. In *Bergey's Manual of Systematic Bacteriology, 2nd edn, vol 3, The Firmicutes*, pp. 1013-1015. Edited by P. De Vos, G. M. Garrity, D. Jones, N. R. Krieg, W. Ludwig, F. A. Rainey, K.-H. Schleifer & W. B. Whitman. New York: Springer.

Song, Y. L., Liu, C. X., McTeague, M., Summanen, P. & Finegold, S. M. (2004). *Clostridium bartlettii* sp. nov., isolated from human faeces. *Anaerobe* **10**: 179-184.

Tamura, K. & Nei, M. (1993). Estimation of the number of nucleotide substitutions in the control region of mitochondrial DNA in humans and chimpanzees. *Mol Biol Evol* **10**: 512-526.

Tamura, K., Stecher, G., Peterson, D., Filipski, A. & Kumar, S. (2013). MEGA6: Molecular Evolutionary Genetics Analysis version 6.0. *Mol Biol Evol* **30**: 2725-2729.

Tan, H. Q., Wu, X. Y., Zhang, X. Q., Wu, M. & Zhu, X. F. (2012). *Tepidibacter mesophilus* sp. nov., a mesophilic fermentative anaerobe isolated from soil polluted by crude oil, and emended description of the genus Tepidibacter. *Int J Syst Evol Microbiol* **62**: 66-70.

Tushar, L., Sasi Jyothsna, T. S., Sasikala, C. & Ramana, C. V. (2015). Draft genome sequence of antimicrobial-producing *Clostridium* sp. JC272, isolated from marine sediment. *Genome Announc* **3**: e00650-00615.

Urios, L., Cueff, V., Pignet, P. & Barbier, G. (2004). Tepidibacter formicigenes sp. nov., a novel spore-forming bacterium isolated from a Mid-Atlantic Ridge hydrothermal vent. *Int J Syst Evol Microbiol* **54**: 439-443.

Varghese, N. J., Mukherjee, S., Ivanova, N., Konstantinidis, K. T., Mavrommatis, K., Kyrpides, N. C. & Pati, A. (2015). Microbial species delineation using whole genome sequences. *Nucleic Acids Res* **43**: 6761-6771.

Wang, Y., Song, J., Zhai, Y., Zhang, C., Gerritsen, J., Wang, H., Chen, X., Li, Y., Zhao, B. & other authors (2015). *Romboutsia sedimentorum* sp. nov., isolated from an alkaline-saline lake sediment and emended description of the genus *Romboutsia*. *Int J Syst Evol Microbiol* **65**: 1193-1198.

Whitehead, T. R., Cotta, M. A., Falsen, E., Moore, E. & Lawson, P. A. (2011). *Peptostreptococcus russellii* sp. nov., isolated from a swine-manure storage pit. *Int J Syst Evol Microbiol* **61**: 1875-1879.

Wong, Y. M., Juan, J. C., Gan, H. M. & Austin, C. M. (2014). Draft genome sequence of *Clostridium bifermentans* strain WYM, a promising biohydrogen producer isolated from landfill leachate sludge. *Genome Announc* **2**: e00077-00014.

Xin, B., Tao, F., Wang, Y., Gao, C., Ma, C. & Xu, P. (2013). Genome sequence of *Clostridium butyricum* strain DSM 10702, a promising producer of biofuels and biochemicals. *Genome Announc* **1**: e00563-00513.

Yilmaz, P., Parfrey, L. W., Yarza, P., Gerken, J., Pruesse, E., Quast, C., Schweer, T., Peplies, J., Ludwig, W. & other authors (2014). The SILVA and "All-species Living Tree Project (LTP)" taxonomic frameworks. *Nucleic Acids Res* **42**: D643-648.

Yutin, N., Puigbo, P., Koonin, E. V. & Wolf, Y. I. (2012). Phylogenomics of prokaryotic ribosomal proteins. *PLoS One* **7**: e36972.

Yutin, N. & Galperin, M. Y. (2013). A genomic update on clostridial phylogeny: Gram-negative spore formers and other misplaced clostridia. *Environ Microbiol* **15**: 2631-2641.

Zindel, U., Freudenberg, W., Rieth, M., Andreesen, J. R., Schnell, J. & Widdel, F. (1988). *Eubacterium acidaminophilum* sp. nov., a versatile amino acid-degrading anaerobe producing or utilizing H_2 or formate. Description and enzymatic studies. *Arch Microbiol* **150**: 254-266.
